# Quantitative analysis of near-implant magnesium accumulation for a Si-containing coated AZ31 cage from a goat cervical spine fusion model

**DOI:** 10.1186/s12891-018-2027-5

**Published:** 2018-04-04

**Authors:** Fan Zhang, Haocheng Xu, Hongli Wang, Fang Geng, Xiaosheng Ma, Minghao Shao, Shun Xu, Feizhou Lu, Jianyuan Jiang

**Affiliations:** 1 Department of Orthopedics, Huashan Hospital, Fudan University, No.12 Wulumuqi Middle Road, Shanghai, China; 2 Department of Research & Tech, Medtronic Greater China co., LTD, Block 11, No.3000 Long Dong Avenue, Pudong, Shanghai China; 3 The Fifth People’s Hospital of Shanghai, Fudan University, Shanghai, China

**Keywords:** Magnesium, Interbody cage, Degradation, Quantitative analysis, Histological concentration

## Abstract

**Background:**

Magnesium (Mg) released from Mg-based implants degradation is believed to be effective in improving osteogenesis, however, studies focusing on Mg-based interbody cages are limited and fusion success was never reported. As excessive Mg accumulation can inhibit new bone formation, this study is designed to explain the possible reasons for the fusion failure of Mg-based cages by analyzing the relationships between the intervertebral Mg accumulation and the resulting interbody fusion.

**Methods:**

The experimental cage was consisted of magnesium alloy (AZ31) substrate and Silicon (Si) -containing coating. C3/C4 and C5/C6 of 24 goats were implanted with cage or autologous iliac crest bone graft (Control group), which were analyzed at 3, 6, 12, and 24 weeks post-operatively. Intervertebral Mg concentrations, Mg-related Calcium (Ca)/ Phosphorus (P) ratios, radiological evaluations and histological findings were recorded for analyzing the relationships between the three of cage corrosion, Mg accumulation, and interbody fusion.

**Results:**

Intervertebral Mg levels were significantly increased after cage implantation, especially in the areas that were closer to the cages at 3 weeks post-operatively, and these increased concentrations could persist up to 12 weeks post-operatively, indicating a relatively rapid corrosion process. Significantly lower Mg levels were only found at 24 weeks post-operatively, but these levels were still higher than those of the control group. In addition, Mg was found to be widely distributed at the intervertebral space since high Mg concentrations could even be detected at the posterior boundary of the vertebral body. Under this Mg accumulation profile, interbody fusion was not achieved, as indicated by the decreased Ca/P ratios, low CT fusion scores and negative histological results.

**Conclusions:**

Intervertebral excessive Mg accumulation might be the primary reason for interbody fusion failure. Quantitative Mg analysis can offer insight into the association between cage degeneration and biological response.

## Background

Magnesium (Mg) is widely believed to be a potentially ideal bioabsorbable orthopedic material that is superior to traditional metallic and biodegradable implants due to its similar mechanical behavior to that of natural bone, excellent osteoconductive bioactivity, and good biocompatibility and radiolucency [1–5]. However, excessive Mg accumulation caused by rapid implant corrosion can result in severe foreign body responses, tissue irritation, decreased mechanical strength of the new bone, and abnormal calcium (Ca) precipitation [6–8], which can ultimately hinder osteogenesis.

With advances in Mg alloy research, many authors have suggested the importance of the quantitative determination of the spatial Mg accumulation for understanding the degradation process and the interaction between Mg release and biological responses [9]. However, no studies were focused on intervertebral Mg accumulation as most of the previous studies were performed for Mg-based screws [6, 10]. Daentzer reported an AZ31 (Magnesium-Aluminum alloy) cage with poly-ε-caprolactone (PCL) coating in a cervical sheep model but interbody fusion was not realized [4, 5]. Though the authors attributed the fusion failure to the hindering of new bone ingrowth by the PCL coating, we considered that the failure might be due to the excessive intervertebral Mg accumulation: 1) The blood supply environment of the endplate-treated disc space after anterior cervical discectomy and fusion (ACDF) is different from that of cortical bone or cancellous bone, which is believed to influence the corrosion rates of Mg-based implants and the absorption of released Mg ions [11]. 2**)** Differences in the stress stimulation between Mg-based cages and screws/plates also determine the unique characteristics of the resulting cage corrosion rate and intervertebral Mg accumulation [12, 13]. As a result, we decided to conduct a quantitative study of near-cage Mg accumulation using a goat cervical spine fusion model. In our study, AZ31 cages were treated with a newly designed micro-arc oxidation (MAO)-treated silicon (Si)-containing coating to increase the corrosion resistance and bone induction activity [14, 15].

The objectives of the study were to comprehensively understand the corrosion kinetics of the Mg-based cage, and to analyze the process of osteogenesis relative to the intervertebral Mg accumulation profile.

## Methods

### Implant

**Fig. 1:**
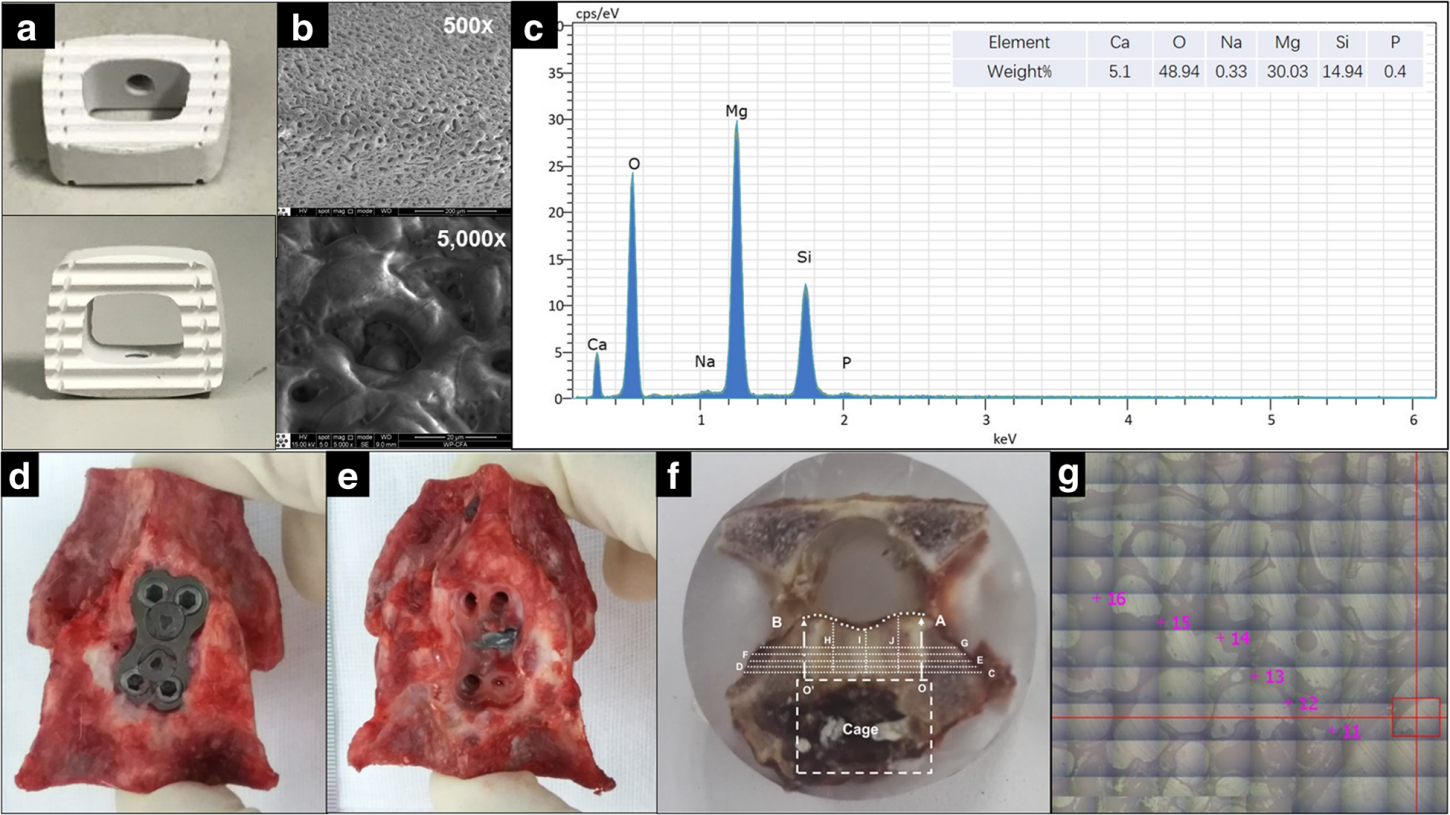
Description of the surface-treated cage and specimens harvested and treated for LA-ICP-MS. **a**, **b**, **c** Morphology of the cage and EDS findings of the coating. **d**, **e** Harvested cervical specimens; **f** C, D, E, F and G are 1 mm, 2 mm, 3 mm, 4 mm and 5 mm from OO’. H, I and J divide line OO’ (and AB) into four equal parts, and all intersection points were targeted as spots for measurements. **g** Point selections by LA-ICP-MS.

The experimental bioabsorbable cage was constructed from the Mg alloy AZ31 (aluminum, 2.5%–3.5%, zinc, 0.6%–1.4%, manganese, 0.2%–1.0%, and Si, maximum 0.3%) in a rectangular design similar to a commercially available graft (Cervios; Synthes, DePuy Spine, Raynham, MA, USA) (Fig. 1a). The Si-containing coating was prepared as previously reported [14–16]. Briefly, the following silicate-based electrolytes were chosen for the MAO treatment: 10 g/l Na_2_SiO_3_·9H_2_O, 1 g/l KOH and 8 g/l KF·2H_2_O. During the MAO process, the applied positive voltage was 460 V, and the pulse frequency was fixed at 600 Hz. The positive and negative duty cycles were 30% and 20%, respectively. The duration of the MAO treatment was 10 min, and the coatings were characterized by scanning electron microscopy (SSX-550) and energy dispersive spectroscopy (EDS) (SHIMADZU, Tokyo, Japan) (Fig. 1b, c).

### Animal work

The animal study was performed after the approval by the Institutional Animal Care and Use Committee (IACUC) at the Department of Laboratory Animal Science, Fudan University (accreditation number 2016-1053-A357). All experimental procedures were performed as described in our previous publication [17]. Briefly, a total of 24 healthy 2-year-old goats (females: 12; males: 12, all of the goats were provided by the Department of Laboratory Science, Fudan University) were randomly assigned to four groups for assessment at 3, 6, 12, and 24 weeks post-operatively. ACDF was performed at C3/4 and C5/6, and one of the two sites was randomly selected for implantation with an AZ31 cage (filled with autogenous iliac bone), while the other was implanted with autologous iliac bone followed by stabilization and titanium plate fixation. The segments with autologous iliac bone grafts served as controls. At each time point (3, 6, 12, and 24 weeks post-operatively), 6 goats were killed by i.v. injection of potassium chloride under pentobarbital sodium anesthesia and the treated segments were harvested for analysis.

### Analysis of Mg concentrations and the related Ca/P(phosphate) ratio

C3/4 and C5/6 motion segments were harvested (Fig. 1d and e) and a laser ablation inductively coupled plasma mass spectrometry (LA-ICP-MS) system was used for the quantitative analysis of Mg, Ca and P. The Ca/P ratio was calculated from the weight% value and reflects the formation of hydroxyapatite (HAP), which is important in osteogenesis, and Ca/P ratio was frequently evaluated in Mg-based implant application [6, 18]. Because excessive Mg is believed to substitute for Ca in HAP structure and result in an inhibitory effect on HAP formation [19–21], osteogenesis could have not been initiated if the Ca/P ratio of the experimental group is much lower than that of the control group.

Specimens were cut along the tangent line of the bony inferior endplate. After appropriate polishing, the specimens were embedded in epoxy resin for LA-ICP-MS analysis. The system comprised a Teledyne Cetac Technologies Analyte Excite laser-ablation system (Bozeman, Montana, USA) and an Agilent Technologies 7700× quadrupole ICP-MS system (Hachioji, Tokyo, Japan). During the measurement, a 193-nm ArF excimer laser, which was homogenized by a set of beam delivery systems, was focused onto the bone surface with a fluency of 7.5 J/cm^**2**^. The ablation protocol employed a spot diameter of 50 μm and a 7-Hz repetition rate for 40 s (equal to 280 pulses). Thirty spots were measured in each specimen, and each spot was located at a fixed distance from the posterior boundary of the cage [1 mm, 2 mm, 3 mm, 4 mm, 5 mm and the posterior boundary of the vertebral body (PB), Fig. 1f and g]. Finally, helium was used as a carrier gas to efficiently transport aerosols to the ICP-MS system for evaluation and the Mg concentration was recorded in parts per million (ppm). United States Geological Survey basaltic glasses (BIR-1G, BHVO-2G, BCR-2G and GSE-1) and the geological apatite standard (Ca_3_[PO_4_]_2_) were used for external calibration. Raw data reduction was performed off-line using a 100% normalization strategy without applying an internal standard.

### Radiographic analysis of cage degradation and interbody fusion

High-resolution CT (interval 0.5 mm; thickness 0.625 mm; 256 slices; Siemens, Erlangen, Germany) was used to quantify cage degradation at the end of each observation period. All images were converted into Mimics 10.0 (Materalise, Leuven, Belgium). An appropriate threshold was selected to outline any cage remaining. Afterwards, the volume of the remaining cage was calculated from a 3D reconstruction, and the decrease in cage volume was calculated based on the volumetric percentage loss. Sagittal CT images were also used to evaluate interbody fusion based on a scoring system reported by Goldschlager et al. (Table 1) [22].

**Table 1:** Scoring System for Computed Tomographic Evidence of Fusion

Score	Description
0	No new bone formation
1	New bone formation but not continuous between C3/4 or C5/6
2	Continuous bridging new bone but comprises <30% of fusion area
3	Continuous bridging new bone but comprises >30% of fusion area

### Histological evaluation

Histological evaluation was conducted as reported previously [4, 23]. Specimens were embedded and polymerized in methyl-methacrylate (Technovit 4000) in accordance with the instructions of the manufacturer. Subsequently, the tissue blocks were further processed using the cutting-grinding technique. The samples were sliced into 300 μm slices and then ground with a plate grinder (EXAKT 400 CS) to a thickness of 10 μm. Sagittal sections of the monosegments in the midline were obtained, stained with toluidine blue according to standard protocols, and then examined by light microscopy focusing. The degree of osteoblast activity and the contact area between the implant surface and the endplate were determined.

### Statistical analysis

Data are presented as ‾x ± s. d. Two-tailed independent t tests were used for comparisons of Mg concentrations, Ca/P ratios and CT fusion scores between the experimental segments and the control group. Differences in Mg concentrations and Ca/P ratios at various distances and time intervals were analyzed by one-way ANOVA followed with an LSD test, which was also used for the comparisons of CT fusion scores at different time intervals. SPSS 19 (IBM Corporation, Armonk, NY) was used for all statistical analyses and a significance level of *P* < 0.05 was chosen.

## Results

### Mg distribution and Ca/P ratio

Mg concentrations and Ca/P ratios of all measured spots are presented in Table 2.

**Table 2:** Relative Mg mass fraction (ppm*10^4^) and corresponding Ca/P ratio

		3 wk	6 wk	12 wk	24 wk
1 mm	Mg	1.48 ± 0.22	1.26 ± 0.15	1.34 ± 0.15	0.85 ± 0.10
	Ca/P ratio	0.77 ± 0.10	1.12 ± 0.21	1.04 ± 0.05	0.97 ± 0.11
2 mm	Mg	1.15 ± 0.08	1.08 ± 0.20	1.08 ± 0.20	0.82 ± 0.06
	Ca/P ratio	0.85 ± 0.06	1.07 ± 0.07	0.98 ± 0.11	1.00 ± 0.12
3 mm	Mg	1.01 ± 0.09	0.87 ± 0.14	1.00 ± 0.14	0.79 ± 0.08
	Ca/P ratio	0.94 ± 0.16	1.15 ± 0.12	1.02 ± 0.09	1.02 ± 0.10
4 mm	Mg	0.99 ± 0.05	0.96 ± 0.06	0.88 ± 0.06	0.83 ± 0.05
	Ca/P ratio	1.00 ± 0.07	1.08 ± 0.05	1.01 ± 0.08	0.96 ± 0.09
5 mm	Mg	0.89 ± 0.14	0.83 ± 0.09	0.89 ± 0.09	0.79 ± 0.07
	Ca/P ratio	1.07 ± 0.07	1.15 ± 0.05	1.09 ± 0.11	1.03 ± 0.08
PB	Mg	0.82 ± 0.12	0.85 ± 0.12	0.88 ± 0.12	0.82 ± 0.05
	Ca/P ratio	1.08 ± 0.05	1.15 ± 0.17	1.09 ± 0.10	0.99 ± 0.1

**Fig. 2:**
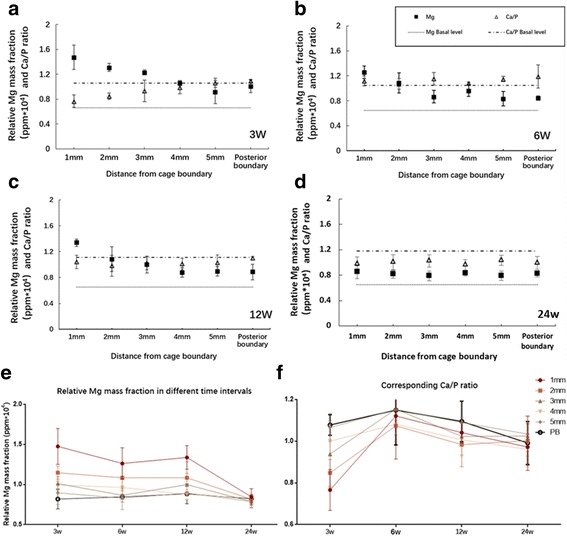
Mg concentrations (recorded in ppm*10^4^) and corresponding Ca/P ratios recorded at 3, 6, 12 and 24 weeks post-operatively are presented. Data represent mean ± s.d, *n* = 3 goats. **a** At 3 weeks post-operatively, Mg concentrations was higher in spots that were closer to the cage and resulted in lower Ca/P ratios. (all P>0.05). **b**, **c** At 6 and 12 weeks post-operatively, Mg concentrations was still higher in spots that were closer to the cage, but Mg began to be uniformly distributed since no differences were observed between those at 4 mm, 5 mm and PB (12 weeks, all P>0.05). Ca/P ratios were uniformly distributed (all P>0.05) and were much lower than the basal level at 12 weeks post-operatively (P<0.001). **d** At 24 weeks post-operatively, Mg was uniformly distributed (*P* = 0.158) but was still higher than the basal level (P<0.001). Ca/P ratios were uniformly distributed (*P* = 0.071) and were much lower than the basal level (*P* = 0.02). **e** Mg was highest at 3 weeks post-operatively, especially in the spots which were more closer to the cage (1 mm, 2 mm, 3 mm, all P<0.05); From 6 weeks to 12 weeks post-operatively, Mg level persisted at a relatively high level; Mg decreased to a significantly low level at 24 weeks post-operatively. **f** Ca/P ratio was the lowest at 3 weeks post-operatively, especially in the spots which were more closer to the cage (1 mm, 2 mm, 3 mm, 4 mm all P<0.05). With the Mg levels decreasing, Ca/P ratios increased but finally decreased to a relatively low level at 12 and 24 weeks post-operatively

Figure 2a shows that the Mg concentration was higher in the spots that were closer to the cage boundary and significantly decreased with increasing distance (3 weeks, *P* < 0.01); No significant differences were observed in the Mg concentration between the spots that were relatively far from the cage at certain time intervals (6 weeks, between 5 mm and the PB; 12 weeks, between 4 mm, 5 mm and the PB, Fig. 2b, c, all *P* > 0.05). At 24 weeks post-operatively, Mg distribution became uniform, as no differences in Mg concentration were found among all of the measured spots (Fig. 2d, *P* = 0.158). The basal Mg concentrations were lower than those in all experimental groups (all *P* < 0.05).

Figure 2e shows that the Mg concentration was highest, regardless of the spot location (all *P* < 0.01), during the initial 3 weeks, except for those at the PB (*P* = 0.192). From 3 to 6 weeks, the Mg concentration began to decrease, but a significant difference was only found for the spots at 1 and 3 mm (all *P* < 0.01). No significant differences in the Mg concentration were observed between 6 and 12 weeks. At 24 weeks post-operatively, the Mg concentration reached a low level (all *P* < 0.05).

In contrast to the high Mg concentrations that were measured in the intervertebral space, the Ca/P ratios of all measured spots at different locations were relatively uniformly distributed in the intervertebral space at 6, 12 and 24 weeks after cage implantation (Fig. 2b, c, d, all *P* > 0.05). Significantly low Ca/P ratios were only observed at 3 weeks post-operatively, especially for spots closer to the cage boundary (1, 2, 3 and 4 mm, all P < 0.01, Fig. 2f). As the Mg concentrations decreased, we observed a steady increase in the Ca/P ratio from 3 to 6 weeks; From 6 to 12 weeks, the Ca/P ratios decreased in most of the measured spots (1 mm, 2 mm, 3 mm and 4 mm, all P < 0.01, Fig. 2f), and a significant decrease in the Ca/P ratio was also observed from 12 to 24 weeks at 4 mm (P < 0.01, Fig. 2f). The Ca/P ratios at 12 and 24 weeks were much lower than the basal level (all P < 0.05) (Fig. 2c, d).

### Radiological analysis of interbody fusion and cage degradation kinetics

**Fig. 3:**
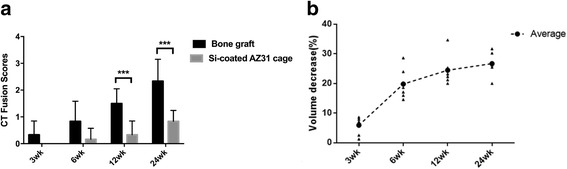
CT fusion scores and cage degradation rate. **a** CT fusion scores of the experimental group and the control group. The fusion score of segments with bone graft was increasing over time (*P* < 0.001), and was significantly higher than that of segments with cage at 12 weeks and 24 weeks post-operatively (*P* = 0.0035 and 0.0024). **b** Volume decreases of the cages during degradation

**Fig. 4:**
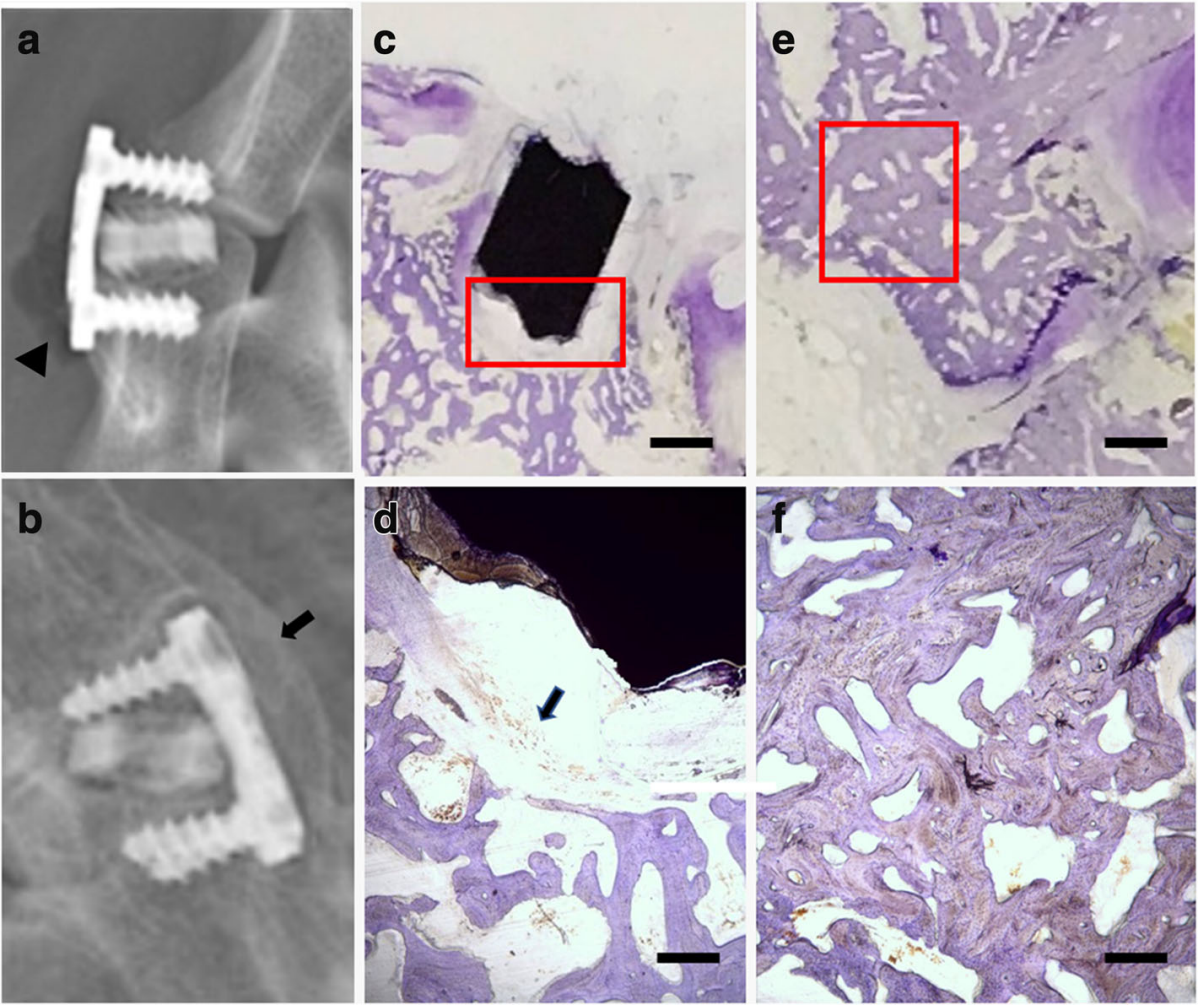
Radiological evaluation and histological findings. The scale bars in **c** and **e** represent 5 mm and the scale bars in **d** and **f** represent 0.5 mm. **a** Gas formation was found. (Triangle). **b** Bone bridges between two vertebral bodies. (Arrow). **c**, **d** No bone tissue was found. Only slight inflammation reaction and fibrous capsule was observed (Arrow). e, f: Successful interbody fusion was observed in the control group

Figure 3a shows that the fusion score of the control group increased over time (*P* < 0.001) and was higher than that of the experimental group at all time intervals (especially at 12 and 24 weeks post-operatively, *P* = 0.0035 and 0.0024). No significant differences were observed in the fusion score increases from week 3 to week 24 in the experimental group (*P* = 0.4), indicating an unsuccessful bony fusion between the vertebral bodies, although bone bridges anterior to the implants were formed by osteophytes at 12 and 24 weeks (Fig. 4b). Moreover, obvious gas accumulation was observed during the first 6 weeks after cage implantation (Fig. 4a).

The degradation kinetics of the Mg structure of the cage is showed in Fig. 3b. At 3 weeks post-operatively, the average volume decrease of Mg was 5.9 ± 3.2%; at 6 weeks post-operatively, the decrease was 19.82 ± 5.40%; at 12 weeks post-operatively, the decrease was 24.47 ± 5.3%; and at 24 weeks post-operatively, the decrease was 26.72 ± 4.11%.

### Histological results

The histological analysis at 24 weeks post-implantation is shown in Fig. 4c-f. Successful bony fusion could be observed in the control group, as a continuous trabecula was found between the endplate and the implanted autogenetic iliac bone (Fig. 4e, f). However, in the experimental group, the remaining cage could still be observed, and the neighboring bone tissue surrounding the cage seemed inconspicuous; that is, neither necrotic foci nor regeneration of bone mass were present, with only fibrous tissue and slight inflammation (Fig. 4c, d).

## Discussion

Although the benefit of Mg for osteogenesis has been well-established by many authors [14, 24–26], opposite conclusions were also suggested by other authors who stated that excessive Mg release would certainly hamper the osteogenic differentiation of stem cells, hinder the proliferation and adhesion of osteoblasts, and even exhibit cytotoxicity [7, 8, 27, 28]. As a result, Mg-based implants should exhibit an appropriate corrosion rate such that the local Mg increase and regional tissue absorption are well balanced. However, few studies have examined the elemental distribution in neighboring bony tissues during the process of implant degradation. Consequently, the maximum Mg levels, the extent of high Mg concentrations and the duration of high Mg concentrations remain unclear, therefore, the quantitative analysis of histological Mg distributions is of great importance for better understanding the implant degradation behavior and the related biological response.

Based on our results, increased accumulation of Mg could remain up to 12 weeks post-operatively, especially at spots closer to the cage during the initial 3 weeks. This result was supported by the facts that the cage volume decreased up to 25% during the first 12 weeks, which was greater than that found during the latter 12 weeks, and obvious gas accumulation was also found at 3 and 6 weeks post-operatively. Excessive histological Mg accumulation was also suspected because the basal levels of Mg were much lower than those in all experimental groups. Those results indicated that though the general cage corrosion rate was not fast, the intervertebral Mg level was still relatively high, and interbody fusion was not achieved under the Mg release profile measured in our study, as indicated by the following two results:

First, our study demonstrated that high Mg concentrations contributed to a steady decrease in the Ca/P ratio, indicating that Ca was partially substituted by Mg during the re-mineralization process, which is consistent with the generally accepted theory that high Mg concentrations can inhibit the precipitation of Ca-containing minerals [23]. Furthermore, the Ca/P ratio ultimately achieved low levels at 12 and 24 weeks post-operatively compared with those in the control group.

Second, the radiological evaluation results showed that although a connected bone bridge was found between the vertebral bodies at 12 and 24 weeks postoperatively (Fig. 4b), the results of CT fusion analysis and histological findings revealed that this did not reflect the situation inside the operated disc space because the bony connections were probably formed by osteophytes in the peripheral areas.

It is interesting to consider why Mg levels released from cages were excessively high and ultimately inhibited osteogenesis because the same implant has been reported to be effective in promoting new bone formation in long bones [15]**.** In fact, since the inflammation initiated by the blood that infiltrates into the implant is the key process of bone repair, the blood supply of the endplate-treated interbody space should be greater than that of the long bone to achieve a more rapid process of histological Mg absorption (bone nonunion is generally more common than interbody fusion failure) [29–31]. As a result, we presumed that the MAO/Si coating might still not produce sufficient effects though the general cage corrosion rate was not fast. We also inferred that stress loaded onto the Mg-based cage might play a key role in accelerating the corrosion process, indicating that an anatomical design might be effective in improving cage corrosion resistance [32].

In addition, a wide distribution of high Mg accumulation was observed in our study, since high Mg concentrations could be detected in areas more than 5 mm from the implant boundary. Considering the close relationship exists between the nerve root and the intervertebral space [33], it may be beneficial if distance is maintained between neural structures and areas with high Mg accumulation because the neuronal cytotoxicity of excessive Mg has been demonstrated by many authors [34].

This study has several limitations. First, the Ca/P ratio was temporarily increased and sometimes was even higher than the basal level, which would have been impossible if the process of new bone formation was not initiated. We suspected that this might be due to Ca release during the degradation of the coating, which contained 5.1% Ca. The use of a Ca-deficient coating would be beneficial for future studies to obtain a more precise conclusion. Second, although we presumed that excessive intervertebral Mg accumulation might be due to the relatively rapid cage degradation during the first 12 weeks, the general cage corrosion rate was not fast, as a result, further studies are still needed to explore why Mg tend to accumulate in the intervertebral space. Finally, because our study is the first one trying to explain the profile of intervertebral Mg distribution, there are lots of key questions need to be answered and one of them is the Mg accumulation on the disc space surrounding tissue, as a result, more studies should be done in the future.

## Conclusions

In conclusion, this study is the first to explain the reasons for the fusion failure of Mg-based cages. Quantitative analysis of near-implant Mg distribution can facilitate an understanding of the association between cage degradation and osteogenesis. The results showed that Mg-based cage may present unique corrosion behavior and excessive intervertebral Mg accumulation caused by the relatively rapid cage degeneration may explain the fusion failure. The corrosion kinetics and osteogenesis inductivity of Mg-based cages should be improved in the future.

## Abbreviations

ACDF: Anterior cervical discectomy and fusion
Ca: Calcium
EDS: Energy dispersive spectroscopy
HAP: Hydroxyapatite
i.v.: Intravenous injection
LA-ICP-MS: Laser ablation inductively coupled plasma mass spectrometry
MAO: Micro-arc oxidation
Mg: Magnesium
P: Phosphate
PCL: Poly-ε-caprolactone
Si: Silicon
